# Character State Reconstruction of Call Diversity in the Neoconocephalus Katydids Reveals High Levels of Convergence

**DOI:** 10.1371/currents.tol.0c5d76728d73ef9c3dbe8065f70ea4cb

**Published:** 2016-03-11

**Authors:** Katy Frederick, Johannes Schul

**Affiliations:** Biological Sciences, University of Missouri, Columbia, Missouri, USA; Biological Sciences, University of Missouri, Columbia, Missouri, USA

## Abstract

The katydid genus *Neoconocephalus* is characterized by high diversity of the acoustic communication system. Both male signals and female preferences have been thoroughly studied in the past. This study used Bayesian character state reconstruction to elucidate the evolutionary history of diverse call traits, based on an existing, well supported phylogenetic hypothesis. The most common male call pattern consisted of continuous calls comprising one fast pulse rate; this pattern is the likely ancestral state in this genus. Three lines of call divergence existed among the species of the genus. First, four species had significantly slower pulse rates. Second, five species had alternating pulse periods, resulting in a double pulse rhythm. Third, several species had discontinuous calls, when pulses were grouped into rhythmically repeated verses. Bayesian character state reconstruction revealed that the double-pulse pattern likely evolved convergently five times; the slow pulse rate also evolved four times independently. Discontinuous calls have evolved twice and occur in two clades; each of which contains reversals to the ancestral continuous calls. Pairwise phylogenetically independent contrast analyses among the three call traits found no significant correlations among the character states of the different traits, supporting the independent evolution of the three call traits.

## Introduction

One of the most intriguing open questions in biology is, how did the diversity of species, forms, adaptations and behaviors that we find today evolve? In particular, how do novel traits evolve, i.e. traits without obvious precursors? Acoustic communication systems with their diversity of call patterns and call recognition mechanisms (i.e., call preferences) have long served as a model for this question (reviews in 1 ^,^ 2)

Communication signals often account for a large part of the phenotypic diversity among closely related species^3^^,^ 4. The rapid diversification of communication systems^5^ enables them to play a major role in speciation; in turn, speciation might also be a driver for the diversification of communication (e.g. 6 ^,^ 7). Signal diversity may be under selection from the environment, and/or constrained by many biological factors^3^^,^ 8. This complexity insures that the diversification of communication systems remains a controversial and important issue.

Understanding the processes that generate and maintain phenotypic diversity requires a solid hypothesis of phylogenetic relationships and knowledge of the variation in the trait(s) under consideration (e.g. 9). Here we present the character state analysis of diverse call traits in the katydid genus *Neoconocephalus* (Orthoptera, Tettigoniidae), utilizing a well supported phylogenetic hypothesis^10^.

The number of species under consideration here is relatively small compared to similar studies (e.g. 11 ^,^ 12). However, a plethora of data exists regarding acoustic communication of *Neconocephalus* and this group has long been established as model for novel trait evolution (reviews in 13 ^,^ 14). In this system males produce calls by rubbing their forewings together, mainly to attract receptive females. The calls are species specific in both temporal and spectral properties. Indeed, the male calls are among the most diverse phenotypic traits in this group and are in many cases the species defining traits. Many aspects of the communication system have been studied: male call pattern (e.g. 15 ^,^ 16 ^,^ 17 ^,^ 18), call mechanics^19^^,^ 20 ^,^ 21 ^,^ 22, female preferences (e.g. 23 ^,^ 24 ^,^ 25 ^,^ 26), and sensory processing (e.g. 27 ^,^ 28 ^,^ 29 ^,^ 30 ^,^ 31). Additionally, a well supported phylogeny for *Neoconocephalus* exists, encompassing most of the Central American, Caribbean and North American species^10^.

Three lines of call diversity exist within this group. Two of them (call structure and pulse pattern) are discrete traits, each with two character states. The third, pulse rate, is quantitative in nature. However, based on the sensory processing of this call character, we treat it here with two character states (fast and slow, see below). Here we present Bayesian character state reconstructions for these three call traits. We then tested for potential links among the diversity of these traits.

## Materials and Methods

We performed a Bayesian character state reconstruction using the *Neoconocephalus* total evidence tree of Snyder et al.^10^ that combined AFLP markers, three nuclear gene markers (ITS1, ITS2, H3) and one mitochondrial gene (CO1). The methodology underlying this tree is given in^10^. The total evidence tree included 17 *Neoconocephalus* species from North and Central America as well as the Caribbean islands. Taxon sampling included all *Neoconocephalus* species occurring in wide geographic ranges. Missing species encompass one endemic species from North America and species endemic to two Caribbean islands. *Bucrates malivolans* and *Belocephalus davisi* were used as out group in 10; these species were not included in the character state reconstruction of this study.

**Character State Reconstruction for discrete Call Traits**

For the three lines of call divergence (see below) we used 10,000 post-burnin trees from the Bayesian total evidence phylogeny to reconstruct the ancestral character states with the program Discrete, implemented in BayesTraits v1.0^32^^,^ 33. This method reconstructs the most probable character states at each tested node, maximizing the likelihood of the character states observed in terminal taxa. Call state reconstructions were completed in batch mode using the concatenated data matrix by running MCMC, 2 million generations 100k burnin, at a 0.2 acceptance rate.

**Phylogenetically Independent Contrasts for Discrete Call Traits**

We used generalized estimating equations (GEE), which handle categorical data and multichotomies found in a tree^34^^,^ 35, to analyze discrete call traits. The compare.gee command requires gee (v.4.13-18^36^), nlme (v3.1-118^37^), and ape (v3.3^38^), packages in R. We ran this analysis using RStudio v0.98.1091 and R v3.1.2. This command runs phylogenetic independent contrasts pair-wise between evolutionary history of the three discrete call traits and the species relationships represented in^10^. These results are then compared to a students t-test to look for significance; additionally, GEE calculates the phylogenetic number of degrees of freedom dfP^34^.

**Call traits and character states of *Neoconocephalus* calls**

The calls of all *Neoconocephalus* species included in this analysis have been described in the literature, as reviewed in^13^, with only a few details added since that time^18^. The published descriptions of the call traits of interest were confirmed during the taxon collection of 10 and during numerous studies of our research group. *Neoconocephalus* calls are produced during opening and closing of the forewings (elytra); loud sound pulses are produced during the closing, while sounds during the opening are typically much softer^22^ and are not required for female responses (e.g.^17^^,^ 18 ^,^ 24 ^,^ 25; but see 26). We therefore limit our description of the temporal pattern to the closing pulses and refer to them simply as 'pulses.' We describe each species’ calls for the three call traits 'call structure', 'pulse pattern', and 'pulse rate.' The character states for these three call traits are listed in Table 1.

**Table 1: Description of Neoconocephalus calls:** The character states for the three call traits of the 17 species considered. C continuous, D discontinuous (=versed) call structure; Pulse rates < 100 Hz are classified as 'slow', > 100Hz as fast. The call structure in *N. ensiger* was equivocal, indicated by a ? (see text); * the verse duration for *N. ensiger* as given here interprets individual pulses as chirps (see text).

Species	Call Structure	Verse Duration	Verse Rate[Hz]	Pulse Pattern	Pulse Rate[Hz]	Source
N. affinis	C	-	-	double	slow 10-12	15 26
N. bivocatus	C	-	-	double	fast 155-175	17 39
N. caudelianus	D	300 ms	1.1	single	fast 192	13
N. ensiger	?	40 ms*	10-15	single	slow 10-14	13 22 27 42
N. exciliscanorus	D	87 ms	3.5	single	slow 83	13
N. maxillosus	C	-	-	double	fast 265-280	15 18
N. melanorhinus	C	-	-	single	fast 140	51
N. nebrascensis	D	1200-1400ms	0.5	single	fast 200	17 22 51
N. palustris	C	-	-	single	fast 225	51
N. punctipes	C	-	-	single	fast 245	15
N. retusiformis	C	-	-	single	slow 49	15
N. retusus	C	-	-	double	fast 140-170	18 22
N. robustus	C	-	-	single	fast 200	17 22 39
N. saturatus	D	50-80ms	2-4	single	fast 161	15
N. spiza	D	50-80ms	2-4	single	fast 153	15
N. triops	D	500-1000ms	1-2	double	fast 180-250	15 22 40
N. velox	C	-	-	single	fast 195	51

The simplest and most common call structure in *Neoconocephalus* consists of pulses produced at a constant, fast pulse rate in the range of 150-250 Hz, produced for extended periods of time (minutes up to hours) without a rhythmic second order time structure. During such calls, the wing movement never stopped at either full closing or full opening^22^; accordingly opening and closing pulses were never separated by more than a few ms. We refer to this call as 'continuous' call structure, with 'single pulse' pattern and a 'fast pulse rate' (Fig. 1A, 3A).

**Pulse Pattern:** Most species of *Neoconocephalus* produce calls that consist of a single pulse rate, i.e. the pulses are evenly spaced (Fig. 1A). In some species, however, shorter and longer pulse periods alternate. The resulting pulse pairs or ‘double pulses’ are then regularly repeated at a 'double pulse rate,' which is equivalent to one half of the individual pulse rate (Fig. 1B). The wing movement during one double pulse begins with a complete opening of the wings followed by a partial closing. Wings open then fully again followed by complete closure.

The wing movement does not stop either within the pulse pair, or between pulse pairs in all *Neoconocephalus* species with this pattern, independently of the double pulse rate i.e. no silent intervals longer than a few ms occur in these species. Five species have double pulse pattern (Fig. 1): *N. bivocatus, N. affinis, N. retusus, N. maxillosus, *and* N. triops* 15 ^,^ 18 ^,^ 39.

**Pulse Rate:** Calls of most *Neoconocephalus* species have unusually fast pulse rates for Tettigoniids (150-250/s)^13^. Several species have dramatically slower pulse rates at about 50-80/s (*N. retusiformis, N. exciliscanorus*) or 10-20/s (*N. ensiger, N. affinis*). The fast pulse rates above about 150/s are at or beyond the limit of the temporal resolution for the sensory system and are perceived as continuous signals without amplitude modulation^17^. Thus, small scale changes of pulse rates are unlikely to be important for female preferences. In contrast, the slow pulse rates below 100 pulses/s can be resolved by the sensory system^17^. We chose to treat this trait as discrete: classifying the calls with pulse rates ≤ 100 Hz as 'slow pulse rate' and all other species as ‘fast pulse rate’ (Fig. 2, Table 1). For species with double pulse pattern (see above), we counted the each individual pulse (i.e. two pulses per pulse pair) when calculating the pulse rate. For the treatment of *N. ensiger*, see below.

**Call Structure:** Several species had a distinct second order time structure in their calls: pulses were produced in distinct 'verses' (= chirps, echemes), which were regularly repeated after silent intervals (Fig. 3B). Durations of chirps and silent intervals ranged from 50ms/250ms to 2s/1.5s. In *N. triops*, the silent intervals were short at 50-100ms, compared to verse durations that varied between 500 and 1000ms among populations (Table 1). Calls with such rhythmic second order time structures were classified as 'discontinuous' call structures, while calls without a rhythmic verse pattern, i.e. pulses produced continuously, were classified as 'continuous' call structures (Fig 3C). For the treatment of *N. triops *and* N. ensiger* see below.

***N. triops:*** The range of this species extends throughout the Neotropics into temperate North America. Throughout its range, *N. triops* has a discontinuous call with 500-1000 ms long verses repeated after silent intervals of 50-100ms duration^15^^,^ 22 ^,^ 40. In North America, although the summer generation has the same discontinuous call structure as all other populations, the winter generation expresses an alternative call type, which is continuous^40^^,^ 41. This alternative call type is the result of developmental plasticity (i.e. is environmentally induced) and the same genotypes can express both phenotypes, depending on the environmental conditions. Because the alternative phenotype is limited to extreme environmental conditions in one population, we classified the call of *N. triops* as 'discontinuous.'

***N. ensiger:*** This species produces a slow pulse rate (about 10/s) continuously. In contrast to all other *Neoconocephalus* species considered here, the wing movement stops completely between pulses for more than 30 ms, so that the opening and closing movement of the wings cover only about 1/2 of the wing cycle. Accordingly, silent intervals of 30-40 ms occur between pulses^13^. This is longer than the typical interpulse durations in this genus, but shorter than the silent gaps between verses in species with discontinuous call pattern (Fig 1C). In addition, the within-male variability of pulse rate of N. ensiger's call was much higher (at constant temperature) than that of other *Neoconocephalus* species^42^.

The long silent intervals and the variability in pulse rate raise the question of whether the pulse rate of *N. ensiger* is homologous to the pulse rate of the other species. Alternatively, *N. ensiger's* pulses could represent a 'one-pulse' verse, and the pulse rate may actually be homologous to the verse rate of discontinuous species. As this question cannot be resolved at this time, we considered the call structure of *N. ensiger* as 'unknown' (Fig. 3). The combined duration of opening and closing pulses in *N. ensiger* is around 30-40 ms, which is equivalent to pulse rates of 25-33 Hz. Therefore, the interpretation of this call as "slow pulse rate" is not affected by the question stated above.

## Results

**Pulse Pattern:** The character state analysis revealed the single pulse pattern (Fig. 1) as the likely ancestral state for this call trait (posterior probability [pp] = 0.99). The double pulse pattern appeared at five tips in the tree, supporting convergent evolution of this character. All five state changes were highly supported (≥ 0.99).

**Character states of 'pulse pattern':**
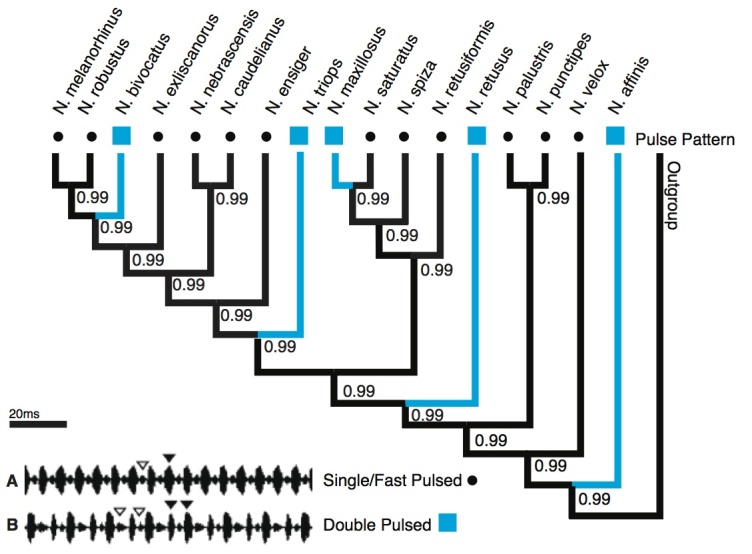
Character state reconstruction of the trait "pulse pattern" of *Neoconocephalus* calls. Call characters of extant species are indicated by shapes at the tips (squares = double pulse, dots = single pulse). Reconstructed character states are represented by branch colors (black = single, blue = double pulse); values at nodes represent posterior probability of character states. **Inset**: Oscillograms of **A** single pulse call, **B** double pulse call.

**Pulse Rate:** This call trait showed a similar pattern as the pulse pattern (Fig. 2). Fast pulse rate was revealed as the ancestral state (pp = 0.99). Slow pulse rate appears at four tips, each with a highly supported character change (pp ≥ 0.99).

**Character states of 'pulse rate':**
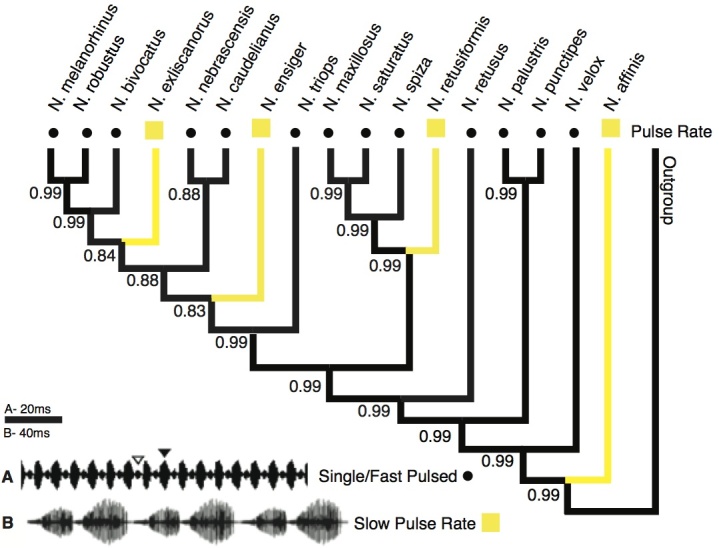
Character state reconstruction of the trait "pulse rate" of *Neoconocephalus* calls. Call characters of extant species are indicated by shapes at the tips (squares = slow, dots = fast). Reconstructed character states are represented by branch colors (black = fast, yellow = slow); values at nodes represent posterior probability of character states. Calls with pulse rates > 140 Hz were classified as 'fast', pulse rates below 100 Hz as 'slow' (see methods). **Inset**: Oscillograms of calls with **A** fast pulse rate and **B** slow pulse rate. The call shown in B is from *N. affinis*, which has double pulse structure; the call of *N. ensiger* shown in Fig. 3C also has slow pulse rate.

**Call structure:** The phylogenetic pattern of this call trait differed from that of the other two traits (Fig. 3). Discontinuous calls occurred in two distinct clades and the character state analysis indicated two separate origins with high posterior probability (pp = 0.99 and pp = 0.98). The ancestral state for *Neoconocephalus* was accordingly a continuous call structure (pp = 0.93). Within each clade with discontinuous calls at least one reversal to continuous calls took place. The treatment of *N. triops* as discontinuous caller did not affect the outcome of this character state analysis: coding this species as 'unknown' or continuous affected only the character state of this species and shifted the character change one node upwards (data not shown).

**Character states of 'call structure':**
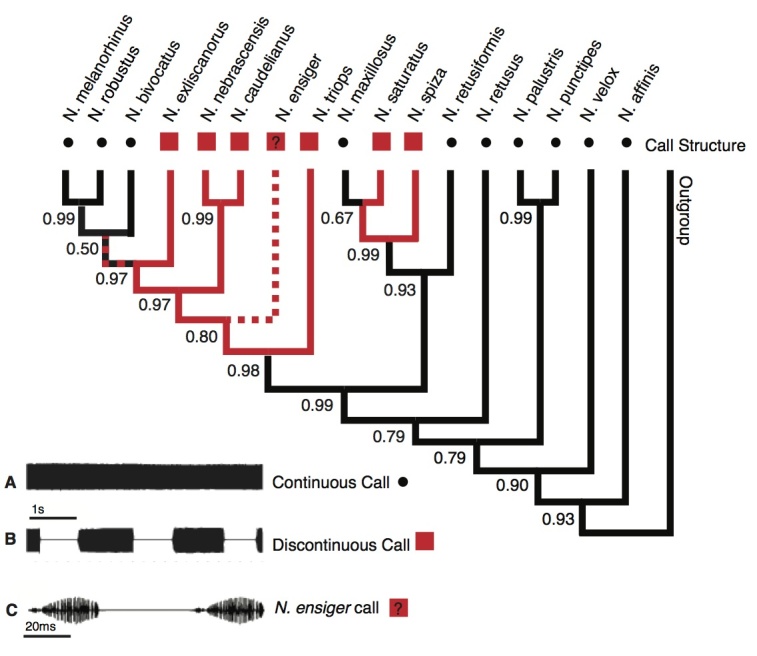
Character state reconstruction of the trait "call structure" of *Neoconocephalus* calls. Call characters of extant species are indicated by shapes at the tips (squares = discontinuous, dots = continuous). Reconstructed character states are represented by branch colors (black = continuous, red = discontinuous); values at nodes represent posterior probability of character states. The red/black dashed line indicates ambiguous character states; the red/white dashed line and ? indicate the unknown character state of the *N. ensiger* call (see methods). **Inset**: Oscillograms of **A** continuous call, **B** discontinuous call, and **C** *N. ensiger* call; note the different time scale in **C**.

**Ancestral call state in *Neoconocephalus***: The character state analyses indicate a continuous, single pulsed call with fast pulse rate as the ancestral call pattern; support for each of these characters was >0.93. This call type occurred in 6 out of 17 extant species; for each individual call trait the ancestral character is more common than the derived character (Figs. 1-3, Table 1).

**Correlation of derived traits**: We conducted pairwise phylogenetically independent contrasts analyses among the three call traits. None of these comparisons produced a significant correlation between character states of the different traits (Table 2). This supports the conclusion that the three call traits evolve independently.

**Table 2: Phylogenetically independent contrasts among call traits:** Results of pairwise comparison using generalized estimating equations. The coefficient of regression (± s.e.m) is given. Phylogenetic number of degrees of freedom dfp = 4.68.

	coefficient ± s.e.m	p-value
pulse pattern vs. call pattern	0.22 ± 0.33	0.42
pulse rate vs. call pattern	0.14 ± 0.34	0.44
pulse rate vs. pulse pattern	0.3 ± 0.27	0.39

## Discussion

The Bayesian character state analysis revealed convergent evolution for the derived states of all three call traits. However, the patterns differed among the three call traits: slow pulse rates and double pulses occurred only at tips of the species tree while the discontinuous call structure occurred in two clades. We found no evidence for correlated evolution among the three call traits.

We identified the ancestral call state of *Neoconocephalus* calls as a continuous call with fast pulse rate and single pulse pattern. Ancestral call structure and pulse pattern are less complex than their derived character states. Calls are produced by rubbing the forewings together; the central pattern generator (CPG) producing the rhythmic pulse pattern was likely derived from the wing beat generator^43^, which typically produces a rate between 10 and 30 Hz (= wing-beat frequency during flight). The ancestral pulse rate in *Neoconocephalus* is derived (and highly unusual) for katydids, where pulse rates are typically below 50-60 Hz^44^. The closest relative of the new-world genus *Neoconocephalus* is the old-world genus *Ruspolia*; indeed, *Neoconocephalus* is likely a monophyletic group inside of *Ruspolia* 13. The most prevalent call pattern in *Ruspolia* is similar to the ancestral *Neoconocephalus* call, with pulse rates around 100 Hz (i.e. slower than in *Neoconocephalus*, yet still fast for katydids) 44 ^,^ 45 ^,^ 46.

In four species, pulse rate reverted back towards slower values. These slow amplitude modulations (AM) are important for female preferences in *N. affinis *and* N. ensiger* 26 ^,^ 42; *N. retusiformis* calls have a distinct slow AM rate, but we lack data on female preferences. Male *N. exciliscanorus* typically have seven opening and closing movements in each chirp^22^. A pulse pattern, however, is not reliably detectable in the resulting sound. Females do not require a pulse pattern for phonotaxis and respond to chirps without internal AM (KHF & JS, unpublished). The differences in motor patterns resulting in slow rates make it unlikely that similar neural or genetic changes underlie the pulse rates in these four species.

Double pulses are a common pattern in katydid calls, occurring in numerous genera across the different Tettiogniid subfamilies^14^^,^ 44. Double pulse pattern in *Neoconocephalus* is unusual in that the wing movement never stops^22^ and no silent gaps longer than a few ms occur. This is true even in the slow calls (pulse rate 10-12 Hz) of *N. affinis*. In all other double pulsing katydid species where data on wing movements are available, the wings come to a complete stop in the closed position^44^, resulting in longer silent gaps between double pulses. The unusual motor pattern in *Neoconocephalus*, and its consistency among the five species, suggests that similar neural changes underlie the independent origins of double pulses in this group. The distribution of double pulses suggests that this pre-disposition was present basally in the *Neoconocephalus* clade.

The discontinuous call structure represents a second order time structure modulating the pulse pattern. This most likely requires the integration of a second pattern generator into neural call production. The CPG generating the breathing rhythm is a likely candidate for modulating Orthopteran calls 47,48. Discontinuous calls occurred in two clades, most likely with independent origins. Character state analysis revealed reversals to the ancestral continuous call structure within each clade. In one clade, both species with discontinuous calls (*N. saturatus, N. spiza*) have very similar verse structure (verse duration 50-80ms, verse rates of 2-4 Hz). In the other clade, verse structure is much more variable (Table 1) across species. Whether similar genetic changes underlie the reorganization of the pattern generator (*i.e.* the inclusion of a second order time structure) is not clear at this time.

We detected no correlations among the character states of the three call traits. This suggests that the neural changes underlying these character states were independent from each other. Accordingly, there are species with a single derived call trait and others with multiple derived traits (*N. affinis*: double pulses & slow pulse rate; *N. triops*: double pulses & discontinuous calls).

Female call recognition mechanisms have been characterized by identifying which combination of call parameters were attractive for females of numerous species (review in 14). In species with ancestral call pattern, female recognition relies on the absence of detectable silent intervals: gaps longer than a few ms render the calls unattractive^17^. No specific AM besides the absence of gaps was required. This mechanism occurs in all species with the ancestral call (*N. robustus* 17,* N. palustris, N. punctipes*; Schul, unpublished data) tested so far. Species with discontinuous calls and the ancestral fast single pulse pattern used the same recognition mechanism to detect pulse pattern, in addition to a verse pattern recognizer^25^^,^ 49. Surprisingly, even two species with the derived double pulse pattern (*N. retusus, N. maxillosus*) continue to use the ancestral mechanism to recognize the pulse pattern, and, accordingly, exhibit no preference between conspecific and ancestral (= single pulse) pattern^18^. Three species with double pulses had call recognition mechanisms that distinctly differed from the ancestral state and from each other^17^^,^ 26 ^,^ 40. One species with slow pulse rate (*N. ensiger*) had another, distinctly different pulse pattern recognizer^42^.

We and others^1^^,^ 15 found an interesting distribution of derived call traits across habitats. Up to four species occur within one habitat and can be heard at the same time; a fifth species may occur asynchronously, during a different season or time of day. Among the four synchronously signaling species is one with the ancestral call pattern, and one for each derived call state. For example, in central Missouri, *N. robustus* (ancestral state), *N. bivocatus* (double pulses), *N. ensiger* (slow pulse rate) and *N. nebrascensis* (discontinuous) signal simultaneously; *N. retusus* occurs and signals later in the season. In Trinidad, a similar acoustic community is composed of different species: *N. punctipes* (ancestral), *N. triops* (double pulses), *N. saturatus* (discontinuous), *N. affinis* (slow pulse rate) and *N. maxillosus* (different activity period, later at night). Accordingly, pairs of sibling species have similar calls, if they do not co-occur (e.g. *N. palustris* occurs in temperate North America, *N. punctipes* in Central America and Caribbean; *N. melanorhinus* is limited to salt marshes, where *N. robustus* does not occur). Groups of closely related species that occur sympatrically have diverse call types.

Replicated evolution among species communities, such as the pattern of derived call traits in *Neonocephalus*, is a key feature of adaptive radiations^50^. The pattern in *Neoconocephalus* suggests that the call diversity and the ability to discriminate among them limits the number of species at each locale. Despite the large quantitative differences within the derived call traits (e.g. chirp durations), two species with the same derived trait do not co-occur. The only exception that we are aware of is *N. triops *and* N. affinis*, which are both double pulsed. However their calls also differ in call structure and pulse rate (Table 1), so they do not occupy the same acoustic niche. These acoustic communities suggest that the sensory processing of the calls plays an important role in the stabilization of derived call traits.

The question of which evolutionary mechanisms drive the diversification of the communication in Neoconocephalus cannot be clearly resolved at this time. We have argued, based on physiological, ecological, and phylogenetic data, that genetic drift might play an important role in call diversification^14^. Data on the age of this group and the derived call traits as well as additional physiological data might inform this question in the future.

## Competing Interests

The authors have declared that no competing interests exist.
